# Colorimetric Method for the Estimation of Escitalopram Oxalate in Tablet Dosage Form

**DOI:** 10.4103/0250-474X.65011

**Published:** 2010

**Authors:** T. Vetrichelvan, K. Arul, M. Sumithra, B. Umadevi

**Affiliations:** Department of Pharmaceutical Analysis, Adhiparasakthi College of Pharmacy, Melmaruvathur-603 319, India; 1 Department of Pharmaceutical Chemistry, College of Pharmaceutical Sciences, Medical College, Thiruvananthapuram-695 011, India

**Keywords:** Escitalopram oxalate, colorimetry, validation

## Abstract

A colorimetric method for the analysis of escitalopram oxalate in pure form and in tablets has been developed based on the formation of chloroform soluble ion associates with bromocresol green acidic dye. The extract of ion associates exhibited absorption maxima at 417 nm obeying Beer's law in the range of 2-10 µg/ml. The method is simple, precise and accurate with recovery of 98-102% and does not require any separation of soluble excipients from tablet dosage form.

Escitalopram oxalate is a newer antidepressant used for the treatment of panic disorder[1,2]. Escitalopram oxalate is S(+) enantiomer of the racemic bicyclic phthalene derivative of citalopram, which is chemically S(+)-1-[3(dimethylamino)propyl]-1-p-flurophenyl-5-phthalene carbonitrile. As it is not official in any Pharmacopoeia, there is no official method for its estimation. HPLC[3–8] methods were reported for the estimation of escitalopram oxalate in biological fluids. The present work describes a simple colorimetric method.

A Shimadzu 1700 Double beam UV/Vis spectrophotometer with 1 cm matched quartz cells was used for absorbance measurements. Tablets-A (Talopam, Lupin Pharmaceuticals Ltd., Mumbai, India), Tablets-B (Celepra, Micro Labs (P) Ltd., Bangalore, India) and Tablets-C (Zetalo, Piramal Health Care, Mumbai, India) were used in this study. Escitalopram oxalate tablets are available in various strength (5, 10 and 20 mg) and procured from a local pharmacy.

Escitalopram oxalate contains a tertiary amino group in its side chain, which readily complexes with an anionic species, bromocresol green (BCG) in chloroform and the neutral pair complex produced with λ_max_ 417 nm was measured spectrophotometrically.

Escitalopram oxalate (50 mg) was dissolved in 0.2 N HCl (50 ml). From the stock solution, 5 ml was diluted to 50 ml with 0.2 N HCl (100 µg/ml). Into a series of five 10 ml volumetric flasks, aliquots of 1-5 ml were taken and made up to the mark with 0.2 N HCl to produce 10-50 µg/ml. From each, 5 ml was transferred to a separating funnel, adding 10 ml of (BCG) solution and 20 ml of phthalate buffer (pH 3) solution and the yellow colored complex extracted with 10, 10 and 5 ml of chloroform. Combined the chloroform layers and made up to 25 ml with chloroform. Absorbance was measured at 417 nm against chloroform blank. The calibration curve was plotted in the concentration range of 2-10 µg/ml.

Twenty tablets of formulation A, B and C were accurately weighed and powdered well. Powdered tablets equivalent to 10 mg of escitalopram oxalate was transferred into a 100 ml volumetric flask, 25 ml of 0.2N HCl was added and sonicated for a few min. The extraction was repeated three times and all extracts were mixed and made up to 100 ml with the same solvent. The solution was filtered through a Whatmann filter paper No. 41. Aliquots of the clear solution was pipetted out (3 ml) into a 10 ml standard flask and made up to the mark with 0.2N HCl to produce 30 µg/ml. Part of the above solution (5 ml) was transferred to a separating funnel, added 10 ml of dye (BCG) and 20 ml of phthalate buffer (pH 3) to obtain yellow colored complex which was extracted with 10, 10 and 5 ml of chloroform. Combined the chloroform layers and made up to 25 ml with chloroform (6 µg/ml). The above procedure was repeated and the absorbance measurements were made on six replicates for each formulation. The amount of escitalopram oxalate was calculated from the calibration curve.

To the pre-analyzed solution of formulations, a known quantity of standard solution was added and the contents were mixed well. Then added, 10 ml of dye (BCG) and 20 ml of phthalate buffer (pH 3). Finally extracted the yellow colored complex with 10, 10 and 5 ml of chloroform, combined the chloroform layers and made up to 25 ml. Absorbance measurements were made at 417 nm. Amount present was calculated from the slope and intercept and the % recovery was calculated. The method was validated for accuracy, precision, specificity, detection limit, quantitation limit and reproducibility.

The linearity of the escitalopram oxalate with ion-pair reagent was constructed. The optical characteristics are shown in Table 1. The limit of detection and the limit of quantification were determined from the calibration values of six replicates and calculated by using slope and standard deviation. The limit of detection was found to be 0.003450326 and the limit of quantification was found to be 0.010455533.

**TABLE 1:** OPTICAL CHARACTERISTICS OF PROPOSED METHOD

Parameter	Colorimetric method
λ_max_ (nm)	417
Beer's law limit (µg/ml)	2-10
Sandell's Sensitivity^a^	0.013784
µg/cm^2^/0.001 A.U)	
Molar Extinction coefficient	30087.4
(1 mol^−1^ cm^−1^)	
Corrélation coefficient (r^2^)	0.999642
Regression equation y=mx+c	Y = 0.07255 X + 0.0005
Slope (m)	0.07255
Intercept (c)	0.0005
LOD (µg/ml)	0.003450326
LOQ (µg/ml)	0.010455533
Standard Error	0.007088

a Sandell sensitivity (S)=10^−3^/a; S=Number of micrograms of the determinant per ml of a solution having a cross section of 1 cm^2^ and absorbance of 0.001 and a=absorbance of 1 µg/ml solution determined in a cuvette with an optical path length of 1 cm.

The concentration of 6 µg/ml of escitalopram oxalate was selected and quantification in formulation was performed. The formulation Talopam 10 mg, Celepra 5 mg and Zetalo 5 mg were selected for the analysis and the amount present was found to be 10.03-10.30, 4.95-5.06 and 5.01-5.15 mg, respectively. In recovery studies the percentage recovery of Talopam, Celepra and Zetalo were found to be 101.32, 98.80 and 99.28, respectively. The result indicates that the proposed method is accurate, sensitive and easy for the determination of escitalopram oxalate in raw material and dosage form.
